# Depth Attenuation Degree Based Visualization for Cardiac Ischemic Electrophysiological Feature Exploration

**DOI:** 10.1155/2016/2979081

**Published:** 2016-11-27

**Authors:** Fei Yang, Lei Zhang, Weigang Lu, Lei Liu, Yue Zhang, Wangmeng Zuo, Kuanquan Wang, Henggui Zhang

**Affiliations:** ^1^School of Mechanical, Electrical & Information Engineering, Shandong University, Weihai 264200, China; ^2^School of Art and Design, Harbin University, Harbin 150086, China; ^3^Department of Educational Technology, Ocean University of China, Qingdao 266100, China; ^4^Institute of Acoustics, Chinese Academy of Sciences, Beijing 100190, China; ^5^School of Computer Science and Technology, Harbin Institute of Technology, Harbin 150001, China; ^6^School of Physics and Astronomy, University of Manchester, Manchester M139PL, UK

## Abstract

Although heart researches and acquirement of clinical and experimental data are progressively open to public use, cardiac biophysical functions are still not well understood. Due to the complex and fine structures of the heart, cardiac electrophysiological features of interest may be occluded when there is a necessity to demonstrate cardiac electrophysiological behaviors. To investigate cardiac abnormal electrophysiological features under the pathological condition, in this paper, we implement a human cardiac ischemic model and acquire the electrophysiological data of excitation propagation. A visualization framework is then proposed which integrates a novel depth weighted optic attenuation model into the pathological electrophysiological model. The hidden feature of interest in pathological tissue can be revealed from sophisticated overlapping biophysical information. Experiment results verify the effectiveness of the proposed method for intuitively exploring and inspecting cardiac electrophysiological activities, which is fundamental in analyzing and explaining biophysical mechanisms of cardiac functions for doctors and medical staff.

## 1. Introduction

Cardiac diseases have been the leading cause of death and disability in the world. Evidence has shown that functional abnormity of heart such as the heart failure may lead to the severe cardiac problem with increased mortality [1]. Heart failure manifests insufficient blood flow pumped for delivering oxygen, which generally appears as pulmonary edema and cardiogenic shock [2]. Cardiac researchers and medical staffs have put forward methods to analyze cardiac functional mechanism to understand and treat heart failure. Serpooshan et al. [3] analyzed the structure and function of the failing heart using the biomimetic three-dimensional technology to enhance cardiac healing after injury. Namazi et al. [4] presented an unusual case of amyotrophic lateral sclerosis (ALS) and the cardiac failure was diagnosed at the final stage of the ALS disease. Alickovic and Subasi [5] applied dwt and random forests classifier for analyzing the heart arrhythmia. Keller et al. [6] established a heterogeneous electrophysiological and three-dimensional anatomical model of human atria to explore atrial functional mechanism. Brocklehurst et al. [7] implied the discrete element method (DEM) to investigate the electromechanical mechanism for human atrial tissue. Then, mechanical contractions of cardiac tissues and their corresponding electrical waves' conduction were successfully simulated. Salinet Jr. et al. [8] presented spectral analysis techniques to visualize intracardiac atrial fibrillation (AF) electrograms, helping guide catheter ablation procedures. Aslanidi et al. [9] constructed a 3D virtual human atria model using cell electrophysiological data with detailed DT-MRI anatomy, which provides a valuable way for investigating electrophysiological behavior in the arrhythmic atria during AF. Zhong et al. [2] discussed the utilization of extracorporeal membrane oxygenation (ECMO) for cardiogenic shock. Sala et al. [10] presented a new transgenic mouse model of to replicate the clinical findings of heart failure.

Ventricle fibrillation (VF) is a serious cardiac functional abnormality that can lead to myocardial infarction. Zhang and Hancox [12] improved Luo-Rudy ventricular action potential models by integrating I-Kr current and inactivation-deficient I-Kr into the previous model and verified that loss of inactivation of the I-Kr led to QT interval shortening. Adeniran et al. [13] further considered stretch-activated channel current (sac) in the single cell models and then incorporated the models into 3D human ventricular tissue models to explore the Short QT Syndrome (SQTS) which is associated with ventricular arrhythmias and sudden cardiac death. The symptom of ischemic greatly increases the probability of occurrence of ventricle fibrillation. It has important meaning to investigate the intricate mechanisms under an ischemic condition in order to better facilitate therapeutic interventions. Although a vast amount of experimental and clinical data of the ionic, cellular, and tissue substrates has been acquired, the precise cardiac mechanisms of ischemia are not well understood. Therefore, any advances in finding and tracking the pathophysiological feature, especially advances that might help analyze and treat the cardiac ischemia more effectively are of great significance. Trejos et al. [14] proposed a mechanism of automatic detecting ischemic events using ECG signals, which allows a better interpretation of cardiac ischemic behavior and results in an increase in the discrimination capability for ischemia detection. Cimponeriu et al. [15] developed a two-dimensional realistic ventricular tissue model. The capacity of the model in simulating pathological conditions was validated on exploring the determinants of electrocardiographic (ECG) morphology and tracking in the ECG pathologic changes of ischemic heart. The cardiac electrophysiological activity has been proven to be important in analyzing functional mechanisms under cardiac physiological and pathological condition. At present, researches have carried out the study on the modeling and simulation of cardiac ischemia based on the ventricular cell model [16–21]. Ten Tusscher and Panfilov [22] created a human ventricular cell model which contains all major ion channel currents and thus simulated the human cardiac electrophysiological properties in a closer way. Chinchapatnam et al. [23] used a fast electrophysiological (EP) model and proposed an adaptive algorithm to estimate cardiac local conduction velocity and apparent electrical conductivity. The method revealed hidden cardiac parameters and can help guide diagnosis and therapy of human left ventricle arrhythmia. A computational cardiac model was applied to simulate the electrophysiological action of two drugs of amiodarone and cisapride in healthy and ischemic ventricle cells for investigating the pharmacological effects, which is helpful to analyze the underlying arrhythmias mechanisms caused by the two drugs [24]. Lü et al. [25] developed a human ventricular cell and tissue ischemic model. Through the model, the functional consequences and mechanisms underlying the arrhythmias in early acute global ischemia are investigated to analyze the influence of acute global ischemia on cardiac electrical activity and subsequently on reentrant arrhythmogenesis. Lu et al. [26] further developed a 3D human ventricular ischemic model combining a detailed biophysical description of the excitation kinetics of human ventricular cells with an integrated geometry of human ventricular tissue. To analyze the spatiotemporal deformation parameters for the myocardial contraction, Han et al. [27] proposed the visualization tools and a strategy for the automatic detection of dysfunctional regions of cardiac ischemic pathologies, which is proved very useful for quantitatively demonstrating the main properties of the left ventricle myocardial contraction. Shenai et al. [28] presented the visualization of normal and ischemic propagation and found intra-QRS changes in and around the ischemic region, which proved that ischemia may cause depolarization changes detectable by both action potentials and unipolar leads. To exhibit the electrophysiological activities under the physiological and pathological condition within the authentic cardiac structure, Wang et al. presented a multivariate visualization method [29] and Zhang et al. proposed an interactive visualization algorithm [30] to visualize both the anatomical data and the electrophysiological data simultaneously. However, these methods cannot explore the hidden electrophysiological feature of pathological tissue in the 3D space.

In this paper, we proposed a visualization framework, which combines the human cardiac ischemic model with a novel depth weighted optic attenuation model, to inspect the occluded cardiac ischemia information with the complicated context of electrophysiological activities under cardiac ischemic condition. First the human ventricle ischemic data is acquired through the cardiac ischemic model. In the proposed depth weighted optic attenuation model, Euclidean Distance Transform (EDT) of each voxel is computed in the electrophysiological data, that is, the Euclidean distance from each voxel to the ventricle boundary, as the coefficient of the attenuation degree of the voxel. This model makes the voxel which is closer to the boundary of the ventricular tissue have the higher attenuation value. Thus, the region that contains the voxels is more transparent. The hidden feature of interest in the ischemic tissue can be revealed from complex overlapping electrophysiological information by the model. The paper is organized as follows.  Section 2 presents the human cardiac tissue ischemic model and visualization framework which includes a novel depth weighted optic attenuation model construction.  Section 3 provides experimental results and discussions. In Section 3, results of the experiments demonstrate that the method we presented can show the feature of cardiac action potential propagation during ischemia more effectively through surrounding complex information. Finally, our conclusions are given in Section 4.

## 2. Design Materials and Methods

To explore organs of interest from mass of cardiac tissues, Zhang et al. [31–33] proposed approaches for revealing detailed structures and further presented a cardiac visualization system, which can provide the user different levels of cardiac anatomy rendering [34]. Yang et al. [35] designed a multidimensional transfer function for visualizing the multiboundary cardiac volume data. Different from the cardiac anatomy characteristic, electrophysiological activities such as excitation propagation in the various human heart tissues are hard to be observed and analyzed in the 3D space. To address this issue, Zhang et al. proposed a GPU-based high performance wave propagation simulation with fine anatomical structure [36]. Based on their work [11, 37], a GPU-based framework for electrophysiological data simulation and visualization is proposed. To fuse cardiac anatomical and electrophysiological model together, Yang et al. [38] designed the fusion transfer function which demonstrated cardiac electrophysiological activity by adjusting the parameter opacity of transfer function.

However, these methods cannot directly explore those cardiac function features at pathological conditions occluded by the complex biophysical information. In this section, we first induce a human cardiac ischemic model to explore cardiac electrophysiological activity and generate the altered ischemic electrophysiology data. Then 3D Euclidean distance transform is implemented on the data, and the depth weighted optic attenuation model is consequently constructed based on the Euclidean distance transform for revealing the hidden cardiac ischemic action potential propagation feature.

### 2.1. Cardiac Ischemic Electrophysiological Model

To explore the cardiac ischemic feature, in this work, the phase of ischemia is considered in the cardiomyocyte electrophysiological model, which describes the cardiac ischemic action potential (AP) generation through the monodomain reaction-diffusion equation as follows:

(1) ∂Vm∂t=−Iion+IstimCm+∇·D∇Vm,Iion=INa+IK1+Ito+IKr+IKs+ICaL+INaCa+INaK+IpCa+IpK+IbCa+IbNa+IKATP,

where *V*_*m*_ represents transmembrane potential and *t* is the time. *I*_ion_ is the total ionic current depending on the voltage and time and *I*_stim_ indicates the externally applied stimulate current. *C*_*m*_ is the transmembrane capacitance per unit membrane area. *D* is the diffusion tensor for describing the tissue conductivity and ∇ is the gradient operator. The ionic current *I*_*K*(ATP)_ in *I*_ion_ is the ATP sensitive *K* ^+^ current which is calculated by the following equation [17]:

(2) IKATP=Vm−EKK+oK+o,controlnfρ0gATPAm,f=fATPfTfMfN,

where *E*_*K*_ is the potassium ion equilibrium potential which is given by Nerst equation [18]:

(3) $$\begin{array}{c} E_{K}=\frac{RT}{F}\log\left(\;\frac{{\left[K^{+}\right]}_{o}}{{\left[K^{+}\right]}_{i}}\;\right), \end{array}$$

where *f*_ATP_ is the fraction of opened channels and *f*_*T*_ is the temperature dependent factor. *f*_*M*_ and *f*_*N*_ are correction factors caused by intracellular Mg^2+^ ions and intracellular Na^+^ ions. *ρ*_0_ is the open probability of a channel in the absence of ATP. *g*_ATP_ is the gate control variable of adenosine triphosphate (ATP) and *A*_*m*_ represents the ratio of cell membrane surface area and volume.

*f*_ATP_ is a Hill equation:

(4) $$\begin{array}{c} f_{ATP}=\frac{1}{1+{\left({\left[ATP\right]}_{i}/K_{m}\right)}^{H}}, \end{array}$$

where *K*_*m*_ and *H* are the nonlinear function of [ADP]_*i*_:

(5) Km=35.8+17.9ADPi0.256,H=1.3+0.74exp⁡−0.09ADPi,

*f*_*T*_ is described by the temperature effect formula:

(6) $$\begin{array}{c} f_{T}\left(\;T\;\right)=Q_{10}^{\left(T-T_{0}\right)/10}, \end{array}$$

where *Q*_10_, *T*, and *T*_0_ represent the temperature coefficient, absolute temperature, and reference temperature, respectively, and *Q*_10_ = 1.3, *T*_0_ = 36°C. *f*_*M*_ is used to explain the inward rectification of intracellular magnesium ions, which is a Hill equation:

(7) $$\begin{array}{c} f_{M}=\frac{1}{1+{\left[Mg^{2+}\right]}_{i}/K_{h,Mg}}. \end{array}$$

Here *K*_*h*,Mg_ is defined as follows:

(8) $$\begin{array}{c} K_{h,Mg}=K_{h,Mg}^{0}\left(\;{\left[\;K^{+}\;\right]}_{o}\;\right)\exp\left(\;-\frac{2\delta_{Mg}F}{RT}V_{m}\;\right), \end{array}$$

where *δ*_Mg_ = 0.32 and *K*_*h*,Mg_^0^([*K* ^+^]_*o*_) is defined by

(9) $$\begin{array}{c} K_{h,Mg}^{0}\left(\;{\left[\;K^{+}\;\right]}_{o}\;\right)=\frac{0.65}{\sqrt{{\left[K^{+}\right]}_{o}+5}}, \end{array}$$

where *f*_*N*_ is used to explain the inward rectifier ion induced cell Boehner, which is also a Hill equation:

(10) $$\begin{array}{c} f_{N}=\frac{1}{1+{\left({\left[Na^{+}\right]}_{i}/K_{h,Na}\right)}^{2}}. \end{array}$$

Here *K*_*h*,Na_ is defined as follows:

(11) $$\begin{array}{c} K_{h,Na}=K_{h,Na}^{0}\exp\left(\;-\frac{2\delta_{Na}F}{RT}V_{m}\;\right), \end{array}$$

where *δ*_Na_ = 0.35 and *K*_*h*,Na_^0^ = 25.9 mM. The parameter setting in the ischemic model can be found in [18, 19].

The electrophysiological data is acquired by implementing the ischemic model on the Visible Human ventricle data. The value of each voxel in the electrophysiological volume data is the action potential of the cardiac cell under the ischemia condition. Thus, the electrophysiological volume data can represent the ventricle action potential propagation during ischemia.

### 2.2. Euclidean Distance Transform

**Figure 1:**
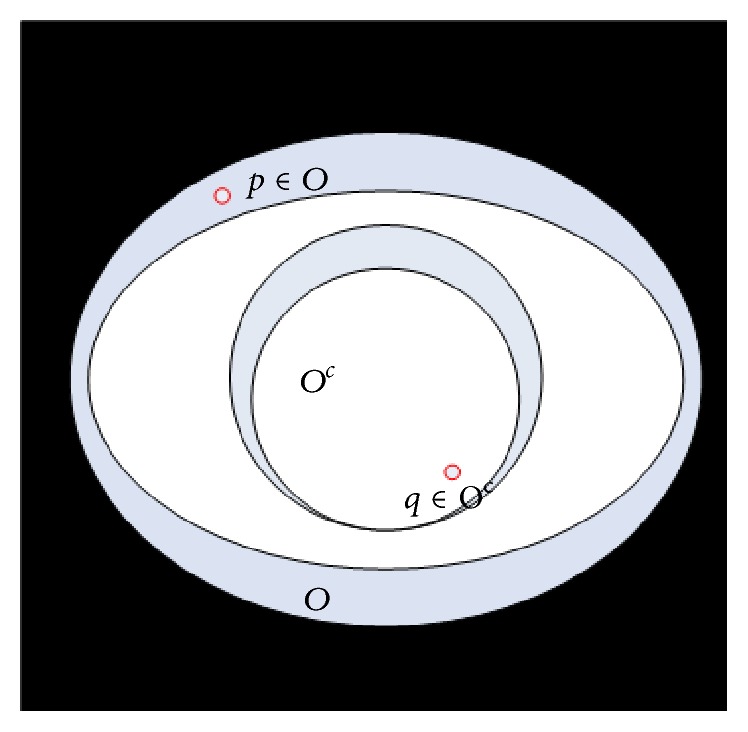
Elements defined in the field of Euclidean distance transform.

Distance transform (DT) maps each point into its smallest distance to regions of interest [39]. The central problem of EDT (Euclidean Distance Transform) is to compute the Euclidean distance of each point to a given subset of a plane. Let *I* : *Ω* ⊂ *Z* ^2^ → {0,1} be a binary image, *Ω* = {0,…, 1} × {0,…, 1}. By convention, 0 is assigned to black and 1 to white. Hence, we have the set *O* which is represented by all white pixels: *O* = {*p* ∈ *Ω*∣*I*(*p*) = 1}, as shown in Figure 1. The set *O* is called foreground and can consist of any subset in the image domain, including disjoint sets. The elements of its complement, *O* ^*c*^, the set of black pixels in *Ω*, are called background. From the DT point of view, the background pixels are called the interest points or feature points.

**Definition 1 .**

The distance transform (DT) is the transformation that generates a map *D* whose value of each pixel *p* is the smallest distance from this pixel to *O* ^*c*^:

(12) Dpmin⁡dp,q ∣ q∈Oc=min⁡dp,q ∣ Iq=0.

*D* is called the* distance map* of* I*. *D* itself can also be called a distance transform. Moreover, *d*(*p*, *q*) is generally taken as the Euclidean distance:

(13) $$\begin{array}{c} d\left(\;p,q\;\right)=\sqrt{{\left(p_{i}-q_{i}\right)}^{2}+{\left(p_{j}-q_{j}\right)}^{2}}. \end{array}$$

To extend the 2D binary image *I* to 3D space, we let *I*_3D_ : *Ω*_3D_ ⊂ *Z* ^3^ → {0,1} be a set of 2D binary images, where *Ω*_3D_ = {0,…, 1} × {0,…, 1} × {0,…, 1}. 0 and 1 are the same as those in 2D binary image. *O*_3D_ and *O*_3D_^*c*^ are object set and the set of black pixels in *Ω*_3D_, respectively. 3D distance map of each pixel *p* in *I*_3D_ is thus defined as

(14) $$\begin{array}{c} D_{3D}\left(\;p\;\right)≔\min\left\{\;d_{3D}\left(\;p,q\;\right)\;|\;q\in {O_{3D}}^{c}\;\right\}, \end{array}$$

and 3D Euclidean distance *d*_3D_(*p*, *q*) is given by

(15) $$\begin{array}{c} d_{3D}\left(\;p,q\;\right)=\sqrt{{\left(p_{i}-q_{i}\right)}^{2}+{\left(p_{j}-q_{j}\right)}^{2}+{\left(p_{k}-q_{k}\right)}^{2}}. \end{array}$$

### 2.3. Depth Weighted Optic Attenuation Model

The optic radiation function for visualizing the cardiac ischemic data acquired by the reaction-diffusion equation in Section 2.1 is [40]

(16) $$\begin{array}{c} C=\overset{D}{\underset{0}{\int }}C\left(\;t\;\right)\tau \left(\;t\;\right)e^{-\int_{0}^{t}\tau \left(s\right)ds}dt, \end{array}$$

where *C*(*t*) is the radiance and *τ*(*t*) is the attenuation degree function of a sample *t* in the cardiac volume data along the view direction.

To inspect the occluded cardiac ischemia information with the complicated context of electrophysiological activities, we consider the calculated 3D Euclidean distance transform of a sample *x*_*i*_ in the cardiac ischemic volume data as the attenuation factor. 3D Euclidean distance transform demonstrates the depth to the boundary of tissues that *x*_*i*_ belongs to. Then the improved depth attenuation degree function can then be acquired as follows:

(17) $$\begin{array}{c} \tau_{depth}\left(\;x_{i}\;\right)=\tau \left(\;x_{i}\;\right)\cdot \Theta_{EDT}\left(\;x_{i}\;\right), \end{array}$$

where Θ_EDT_(*x*_*i*_) is associated with unit normalized 3D Euclidean distance transform result, which is thought to be the depth of *x*_*i*_.

We incorporate the depth attenuation degree function into the optic radiation function and construct the depth weighted optic attenuation model as

(18) $$\begin{array}{c} C=\overset{D}{\underset{0}{\int }}C\left(\;t\;\right)\tau_{depth}\left(\;t\;\right)e^{-\int_{0}^{t}\tau_{depth}\left(s\right)ds}dt, \end{array}$$

where attenuation *τ*(*t*) is replaced by *τ*_depth_(*x*_*i*_). Thus, the opacity of a volume sample will increase when it has a larger depth to the boundary, which means that a sample is more opaque when it is farther from the boundary. The hidden ischemia region of pathological tissue can then be revealed from complex overlapping information generated by the cardiac physiology model.

## 3. Experimental Results

**Figure 2:**
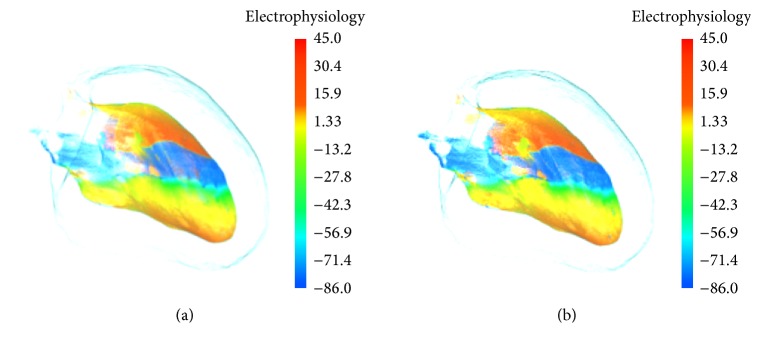
Electrophysiology visualization of inner left ventricle: (a) under the normal condition and (b) under the ischemia condition.

In this section, the proposed depth weighted optic attenuation model was applied on the acquired electrophysiological ischemic data. Then the performance of the visualization method is assessed. The method exploited the visualization toolkit (VTK) libraries and the visualization system was developed under the environment of Visual Studio 2010. In this study, the electrophysiological ischemic model of cardiomyocytes is implemented to describe biophysical properties of the heart under pathological condition. In the simulation, the interior features of acquired cardiac ischemic electrophysiology data are impossible to be explored through traditional optic model.  Figure 2 depicts the traditional electrophysiology visualization result of stimulated inner left ventricle muscles under the normal and ischemic condition using the normal optic radiation model. Conventional visualization of excitation propagation under the normal condition is shown in Figure 2(a).  Figure 2(b) shows excitation propagation under the ischemic condition. Due to the mild ischemia and small ischemic region, the influence on excitation propagation is not able to spread to the surface of inner left ventricle muscles. Action potential propagation on the surface under the ischemic condition thus performs the same as the spiral wave shown under the condition of normal propagation. We can therefore hardly differentiate these two excitations from each other based on the patterns of wave propagation on the surface layer.

**Figure 3:**
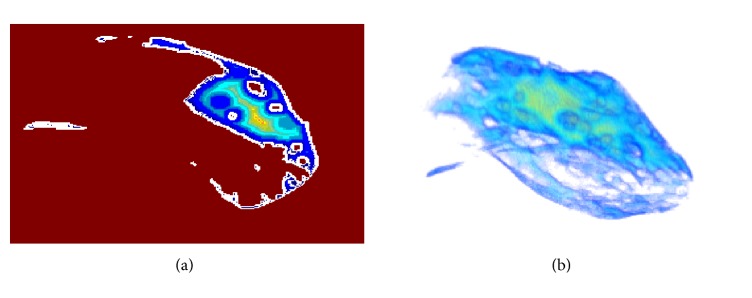
3D Euclidean distance transformation in 3D space of inner left ventricle: (a) 2D projection slice and (b) 3D illustration.

To implement the 3D exact Euclidean distance transform on the electrophysiology volume data, we associate those voxels on the boundary in volume to “black” pixels in distance transform terminology, and voxels inside the material are associated with the “white” pixels. In this way, the depth of inner voxels to boundary is represented by distance transformation of those voxels.  Figure 3(a) shows the effect of 2D projection slice of distance transformation in 3D space of inner left ventricle muscles. The value in each pixel which represents the smallest distance from this pixel to black pixels is mapped onto color which changes from blue to red with increasing of the distance to the boundary. Through the 3D exact Euclidean distance transform, the depth of inner samples in cardiac ischemic data to boundary is represented by distance transformation of those samples.  Figure 3(b) shows the effect of exact Euclidean distance transformation in 3D space of inner left ventricle muscles. The value of each pixel represents the smallest distance from this pixel to the “black” pixels and is mapped onto color, which changes from blue to red with increasing of the distance to the boundary.

**Figure 4:**
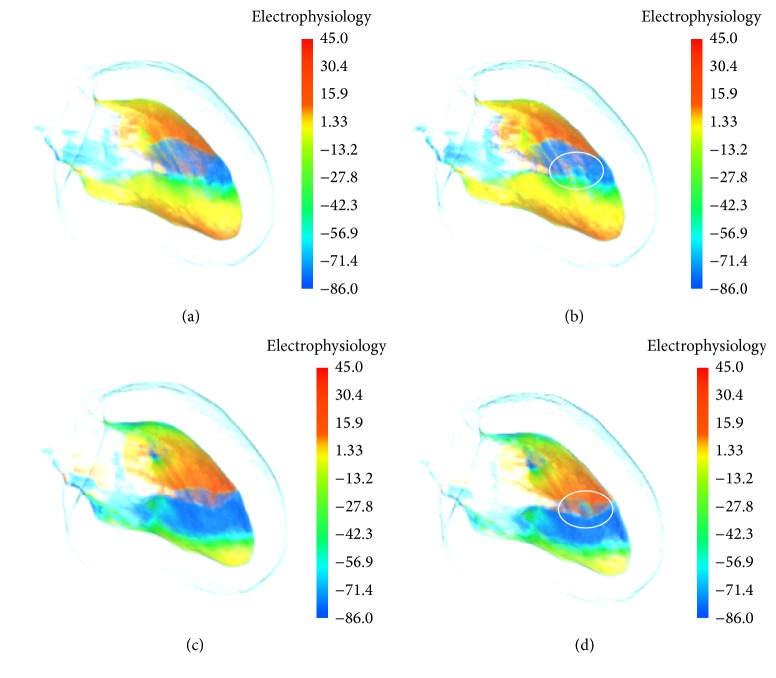
Electrophysiology visualization of inner left ventricle at different time under the ischemia condition: (a) by conventional optic radiation model at 720 ms; (b) by the depth weighted optic attenuation model at 720 ms; (c) by conventional optic radiation model at 1040 ms; (d) by the depth weighted optic attenuation model at 1040 ms.

Figure 4 shows the effect of revealing interior ischemia region at the different time with the proposed depth weighted optic attenuation model. Using traditional optic radiation model, electrophysiology visualization of inner left ventricle muscles at 720 ms under the ischemic condition is demonstrated in Figure 4(a). Since the excitation propagation on the surface layer is the same as the excitation at normal physiological condition, the feature of ischemic electrophysiology activity of left ventricle cannot be distinguished. In Figure 4(b), the electrophysiological information which is mapped onto color gradually fades to transparent with decreasing distance to boundary in the anatomical model. Interior ischemia region is then able to be highlighted from surrounding complex electrophysiological and anatomical context. The region marked in Figure 4(b) in the white ellipse is the myocardial blood clot which is revealed from the occlusion caused by surrounding biophysical activity. As seen in Figure 4(b), since the propagation velocity of the reentrant in the ischemia region slows down, a wavefront gap appears in the result image. At this time, with the propagation of the reentry wave, the conduction block is generated in the region, which will increase the transition probability of ventricular tachycardia to ventricular fibrillation.  Figure 4(c) shows ischemic electrophysiological activity at 1040 ms using traditional optic radiation model. In Figure 4(c), reentrant on the surface propagates forward stably, while the inner feature is occluded due to outer sophisticated overlapping biophysical information. Using the presented depth attenuation degree based model, as marked by the white ellipse in Figure 4(d), the hidden ischemia feature is distinctly explored. From Figure 4(d) we can see that the wave velocity in the ischemic area is slower than that in the surrounding normal tissue and the reentrant attempts to spread through the ischemic area. However, the reentry wave of the surrounding normal tissue continues to spread without conduction block.

**Figure 5:**
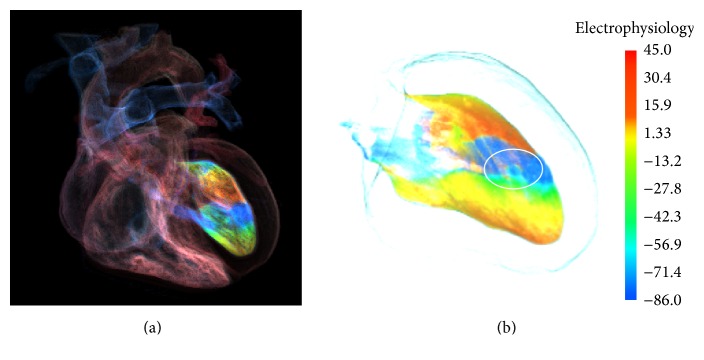
Electrophysiology visualization of inner left ventricle under the ischemia condition: (a) by the GPU-based multimodality simulation [11] and (b) by our proposed method.

Figure 5 shows the GPU-based multimodality simulation in [11] and the effect of exploring cardiac ischemic electrophysiological activity by the method in this work. In Figure 5(a), inner left ventricle muscles under the ischemia condition are incorporated with cardiac anatomy model, while features in ischemic region are occluded by the action potential propagation in the outer tissue. In addition, the rendering result presented in [11] does not provide quantitative information about the excitation propagations. To improve this situation, as shown in Figure 5(b), through the method proposed in this study, the hidden ischemia region and the feature of excitation propagation in the region are revealed clearly in a quantitative way.

## 4. Conclusions

In this paper, we implemented a human cardiac ischemic model and revealed the hidden cardiac biophysical behavior under the ischemic condition by the depth attenuation degree based optic attenuation model. To explore the important features of interest of the heart under the pathological condition of ischemia, we first used a human cardiac ischemic model and acquired cardiac ischemic electrophysiology data. Then the depth of a sample to its boundary in the data is computed through the 3D Euclidean distance transform. We integrated the generated depth attenuation degree function based on the 3D Euclidean distance transform into the normal optic radiation model and then constructed the depth weighted optic radiation model. The experimental results showed that hiding features in ischemia region are effectively explored with complex electrophysiological context, which provides those medical staff and cardiac researchers with new information of the underlying cardiac biophysical mechanisms.
